# Status Epilepticus Triggers Time-Dependent Alterations in Microglia Abundance and Morphological Phenotypes in the Hippocampus

**DOI:** 10.3389/fneur.2017.00700

**Published:** 2017-12-18

**Authors:** Season K. Wyatt-Johnson, Seth A. Herr, Amy L. Brewster

**Affiliations:** ^1^Department of Psychological Sciences, College of Health and Human Sciences, Purdue University, West Lafayette, IN, United States; ^2^Weldon School of Biomedical Engineering, West Lafayette, IN, United States

**Keywords:** status epilepticus, epilepsy, hippocampus, microglia morphology, microglia

## Abstract

Status epilepticus (SE) is defined by the occurrence of prolonged “non-stop” seizures that last for at least 5 min. SE provokes inflammatory responses including the activation of microglial cells, the brain’s resident immune cells, which are thought to contribute to the neuropathology and pathophysiology of epilepsy. Microglia are professional phagocytes that resemble peripheral macrophages. Upon sensing immune disturbances, including SE, microglia become reactive, produce inflammatory cytokines, and alter their actin cytoskeleton to transform from ramified to amoeboid shapes. It is widely known that SE triggers time-dependent microglial expression of pro-inflammatory cytokines that include TNFα and IL-1β. However, less is known in regards to the spatiotemporal progression of the morphological changes, which may help define the extent of microglia reactivity after SE and potential function (surveillance, inflammatory, phagocytic). Therefore, in this study, we used the microglia/macrophage IBA1 marker to identify and count these cells in hippocampi from control rats and at 4 h, 3 days, and 2 weeks after a single episode of pilocarpine-induced SE. We identified, categorized, and counted the IBA1-positive cells with the different morphologies observed after SE in the hippocampal areas CA1, CA3, and dentate gyrus. These included ramified, hypertrophic, bushy, amoeboid, and rod. We found that the ramified phenotype was the most abundant in control hippocampi. In contrast, SE provoked time-dependent changes in the microglial morphology that was characterized by significant increases in the abundance of bushy-shaped cells at 4 h and amoeboid-shaped cells at 3 days and 2 weeks. Interestingly, a significant increase in the number of rod-shaped cells was only evident in the CA1 region at 2 weeks after SE. Taken together, these data suggest that SE triggers time-dependent alterations in the morphology of microglial cells. This detailed description of the spatiotemporal profile of SE-induced microglial morphological changes may help provide insight into their contribution to epileptogenesis.

## Introduction

Status epilepticus (SE) is defined by the occurrence of prolonged “non-stop” seizures that last for at least 5 min (1). In the United States, it is estimated that up to 41 out of 100K individuals are affected by SE, and that one or two SE events can increase the risk of future unprovoked seizures by 40–52 and 73%, respectively (1). SE is a clinical emergency for which rapid intervention can help to prevent or reduce the risk for subsequent neuronal injury and neurological sequelae that includes epilepsy and cognitive disturbances (1, 2). Extensive evidence supports that SE provokes an array of inflammatory responses including activation of microglial cells, the resident immune cells of the CNS, which are thought to contribute to the neuropathology and pathophysiology of epilepsy (3, 4).

Microglial cells are highly dynamic professional phagocytes that resemble peripheral macrophages. Under physiological conditions, these cells occupy non-overlapping territories where they constantly survey their surrounding environment for signals that indicate injury and immune disturbances, as well as altered neuronal activity (5–10). Upon sensing pathological signals, including seizures and SE, microglia become reactive and promptly undergo biochemical changes, producing pro-inflammatory cytokines, such as tumor necrosis factor-alpha (TNFα) and interleukin 1- beta (IL-1β) (4). In parallel, reactive microglia go through morphological changes that range from a phenotype of small cell bodies with vastly ramified processes (surveilling microglia) to small amoeboid shapes with little to no processes (activated/phagocytic) that can alternate between transitional states/phenotypes that include slightly enlarged cell bodies with thickened processes that may be long or short (10–16). Both inflammatory and morphological microglial alterations have been widely described in human epileptic brain tissues (4, 17–21) as well as in animal models of SE and experimental epilepsy (4, 22–28). While these and numerous additional studies support that areas such as the hippocampus are particularly vulnerable to SE-induced injury and show highly activated inflammatory responses, less is known in regards to the spatiotemporal progression of microglial morphological changes. Understanding these phenotypes may help define the extent of microglia reactivity and potential function after SE.

Previously, we reported that SE promoted an increase in the microglial levels in the CA1 hippocampal area that peaked at 2 weeks following the prolonged seizures (22). However, this finding was based on densitometry analysis from the immunoreactivity of the ionized calcium-binding adaptor molecule 1 (IBA1), a protein found specifically in microglia and macrophages (29). Thus, in order to determine the extent to which SE modulates the hippocampal microglial population and morphological phenotypes in the current study, we determined (i) the abundance of microglia by immunohistochemistry (IHC) and flow cytometry and (ii) the percentage of microglial morphological phenotypes (ramified, hypertrophied, bushy, amoeboid, and rod) present in different hippocampal areas at 4 h, 3 days, and 2 weeks following a single episode of SE using the pilocarpine rat model of SE and acquired temporal lobe epilepsy.

## Materials and Methods

### Animals

Male Sprague Dawley rats (150–175 g) (Envigo Laboratories) were housed at the Psychological Sciences Building at an ambient temperature of 22°C, with 12-h light and 12-h dark (0800 to 2000 hours) cycles, and unlimited access to food and water. All animal procedures were approved by the Purdue Animal Care and Use Committee and followed the approved Institutional and NIH guidelines.

### Pilocarpine-Induced SE

Status epilepticus inductions were done following previously described protocols (22, 23). Scopolamine methylbromide (1 mg/kg) was given 30 min (min) prior to injections (i.p.) of saline (Sham-Control) or pilocarpine (280–300 mg/kg; Sigma Chemical Co., St. Louis, MO, USA) (SE group). SE was stopped after 1 h with diazepam (10 mg/kg; i.p.; Sigma Chemical Co.). When rats reached class 5 limbic motor seizures (rearing and falling) (30), they were considered to be in SE. Two hours after SE, all rats were given injections (i.p.) of sterile 0.9% saline (AddiPak) for hydration and as needed thereafter. Rat chow was supplemented with sliced peeled apples and Kellogg’s Fruit Loop cereal for up to 1 week after SE inductions. From our studies in the pilocarpine model of SE and acquired TLE, we calculated that pilocarpine promotes SE at 4.5–6 seizure stages according to the Racine scale (30) in approximately 67.1% of the rats. Rats that did not reach a seizure score of 4.5–6 were not used in this study. Animals for histology were sacrificed at 4 h (*N* = 4), 3 days (*N* = 8), or 2 weeks following SE (*N* = 5); controls (*N* = 5). Animals for flow cytometry were sacrificed at 2 weeks following SE (*N* = 5); Controls (*N* = 5). The sample size per group was determined using a power level of 0.80 and α = 0.05 (*post hoc* power analysis, GPower).

### Flow Cytometry

Animals were anesthetized with beuthanasia (200 mg/kg) and perfused with ice cold 1× phosphate buffered saline (PBS). Hippocampi were rapidly dissected on ice and processed using the NeuroCult™ Enzymatic Dissociation Kit for Adult CNS Tissue (Mouse and Rat) according to manufacturer’s instructions (Stemcell Technologies) with minor modifications. At this point, all samples were coded for experimenter to be blinded of treatment groups for the rest of the tissue processing and flowcytometric analysis. Hippocampal tissue samples were then placed into a 70-µm nylon cell strainer rinsed with 100 µL of cold NeuroCult Tissue Collection Solution and gently pressed, using fingertips, through the filter into the 15-mL tube on ice. The collected tissue homogenate was then similarly gently pressed through a 40-µm nylon cell strainer, collected, and transferred into a 1.5–2 mL tube using a glass Pasteur Pipette. The samples were centrifuged at 100 *g* for 7 min at 4°C. Pellets were suspended with 500 µL NeuroCult Enzymatic Dissociation Solution and incubated for 14 min at 37°C. Then, 500 µL of NeuroCult Inhibition Solution was gently mixed and samples were centrifuged at 100 *g* for 7 min at 4°C. The following steps were done using previously described protocols with minor modifications (31–34). Pellets were suspended with 3.5 mL of resuspension solution and 1.5 mL of Percoll gel for a 30% total Percoll (v/v) and centrifuged at 700 *g* for 11 min at RT. The resulting pellets were suspended in eBioscience Flow Cytometry Staining Buffer according to manufacturer’s instructions. Cells in staining buffer were divided equally into four, 1 mL volumes and the number of viable cells per milliliter was estimated with a hemocytometer and Trypan Blue dye (~1 × 10^6^ cells/mL). Immunophenotyping categories identified microglia/macrophages expressing high or low levels of cluster of differentiation molecule 11b (CD11b), cluster of differentiation 45 (CD45), and major histocompatibility complex class II (MHCII) (10) using the following antibodies: APC/Cy7 anti-rat CD45 (BioLegend Cat# 202216 RRID:AB_1236411), APC anti-rat CD11b/c Antibody (BioLegend Cat# 201809 RRID:AB_313995), and PE anti-rat RT1B Antibody (BioLegend Cat# 205308 RRID:AB_1595483). Antibodies were incubated for 30 min at 2–8°C in the dark followed by centrifugation at 400 *g* for 5 min at RT. Cells were washed and centrifuged at 100 *g* for 7 min, following pellet suspension with the Viability Dye (eBioscience, Grand Island, NY, USA, cat. 65-0863). The dye was titrated and added, immediately mixed, and incubated in the dark at 2–8°C for 30 min. Cells were washed and centrifuged at 400 *g* (2×), and pellets were suspended 1:1 of flow cytometry staining buffer and IC Fixation Buffer (eBioscience, Grand Island, NY, USA, cat. 00-8222). Samples were stored at 2–8°C in the dark and analyzed within 3 days. Stained samples were acquired on a BD Facs Aria flow cytometer (BD Biosciences) fitted with a 355 nm-UV laser, 405-nm violet laser, 488 nm-blue laser, a 561-yellow-green laser, and a 627-nm red laser. Data were acquired using DIVA v. 7 (BD Biosciences, San Jose, CA, USA) software. Analysis of the populations was performed in Flowjo v.8.7.3 (Treestar) using bi-exponential display and was based on “fluorescent minus one” gating controls to ensure the proper identification of true positive and negative events. Live cells were gated for CD45/CD11b positive cells and from these the percent of the population of MHCII-positive cells was determined.

### Immunohistochemistry

Animals and tissues were processed for IHC as described in our previous study (22). The tissues used for IHC and densitometry analysis in Schartz et al. (22) were further analyzed for microglia/macrophage cell and morphology counts in the current study. Briefly, animals were anesthetized with beuthanasia (200 mg/kg) and perfused with ice cold 1× PBS followed by 4% paraformaldehyde (PFA). After overnight post-fixation (4%-PFA) and cryoprotection (30% sucrose), brains were frozen in pre-chilled isopentane and stored at −80°C until used. Coronal brain sections (50 µm) were stored in 1×PBS + 0.1% sodium azide at 4°C. Serial sections from rostral to caudal (4–6 sections per brain) were collected at approximately every 500 µm along the dorsoventral axis between the Bregma coordinates −3.00 mm and −5.28 mm. These sections were immunostained with anti-rabbit IBA1 (1:500; Wako Chemicals Cat# 019-19741, RRID: AB_839504) followed by biotinylated goat anti-rabbit secondary antibodies (1:2,000; Vector Laboratories Cat# BA-1000 RRID:AB_2313606), incubated in ABC avidin/biotin complex solution and developed using the DAB Peroxidase (HRP) Substrate Kit, 3,3′-diaminobenzidine (Vector Laboratories). Brain sections were mounted on gelatin-coated slides, Nissl stained, dehydrated in alcohol, de-fatted in Xylene, and coverslipped using Permount mounting media. All chemicals were obtained from Fisher Scientific unless otherwise indicated.

### Morphological Assessment and Cell Counts

The ionized calcium-binding adapter molecule 1 (IBA1) was used to identify microglia. Note that in addition to its specificity for microglial cells, IBA1 also stains infiltrated macrophages and/or monocytes (29). Therefore, to include the possibility of non-microglia cells stained with IBA1, we refer to the IBA1-positive cells as microglia/macrophages. The morphologies of IBA1-positive microglia/macrophages were sorted into categories from 1 to 5: 1—ramified; 2—hypertrophic; 3—bushy; 4—amoeboid; and 5—rod. Cells were categorized based on overall diameter including processes and evident changes in process thickness. These classification guidelines and categories were adopted based on published literature with detailed descriptions of microglia morphology (10–14, 35–37). The descriptions and cellular diameters for each category are as follows: (1) ramified cells had a diameter of 50+ μm with fine and highly branched processes; (2) hypertrophic cells had a diameter of 40–50 µm with thick and highly branched processes; (3) bushy cells had a diameter of 20–25 µm with thick, dense, and shorter processes; (4) amoeboid cells had a diameter smaller than 10–15 µm with retracted processes and irregular shapes; (5) rod cells did not display circular diameters but had long, slender cell bodies and short fine processes. Although not described in this study, we also observed rod microglia/macrophages on train formation, which included two or more connecting rod-shaped cells (13, 14). Cells were categorized in the hippocampal CA1, CA3, and dentate gyrus (DG). IBA1-positive cells were only counted if more than 75% of their staining was inside the boundaries of the counting frame and met the criteria of one of the different diameters (35). Cell overlapping, broken tissue areas, or cells with very light staining were not counted. Cellular nuclei were identified through Nissl staining. To remain unbiased, often multiple cells were counted as one: (1) if microglia appeared to be in cell division or (2) if clumps of amoeboid-shaped microglia were observed, in this case the distinct cells at the edge of the clump were counted but the center of the mass was counted as one cell. A Leica DM500 microscope with high resolution digital camera (Leica MC120 HD) and LAS4.4 software was used for image acquisition using a 40× objective. All image groups were blinded to the researcher for cell counts and morphological quantification and IBA1-positive cells within the entire 40× image frame were counted. We analyzed 4–6 sections per brain bilaterally distributed from rostral to caudal. Four pictures were taken in non-overlapping locations in each of the different hippocampal areas and the average of cell counts was calculated for section area and then for each brain. Cells were counted using ImageJ with scaled circle frames for diameter reference. Total number of cells per frame was counted first, followed by the count of the different morphologies. The percentage of each morphological phenotype was calculated based on the total number of IBA1-positive cells per hippocampal area.

### Statistical Analyses

Graphpad Prism was used for data analysis. *T*-test or analysis of variance were used to determine statistical associations between the experimental groups from flow cytometry and histological data. Statistical significance was set at α < 0.05. Data values are reported as means (M) ± SEM. Figures were generated using Adobe Photoshop (CS6).

## Results

**Figure 1:**
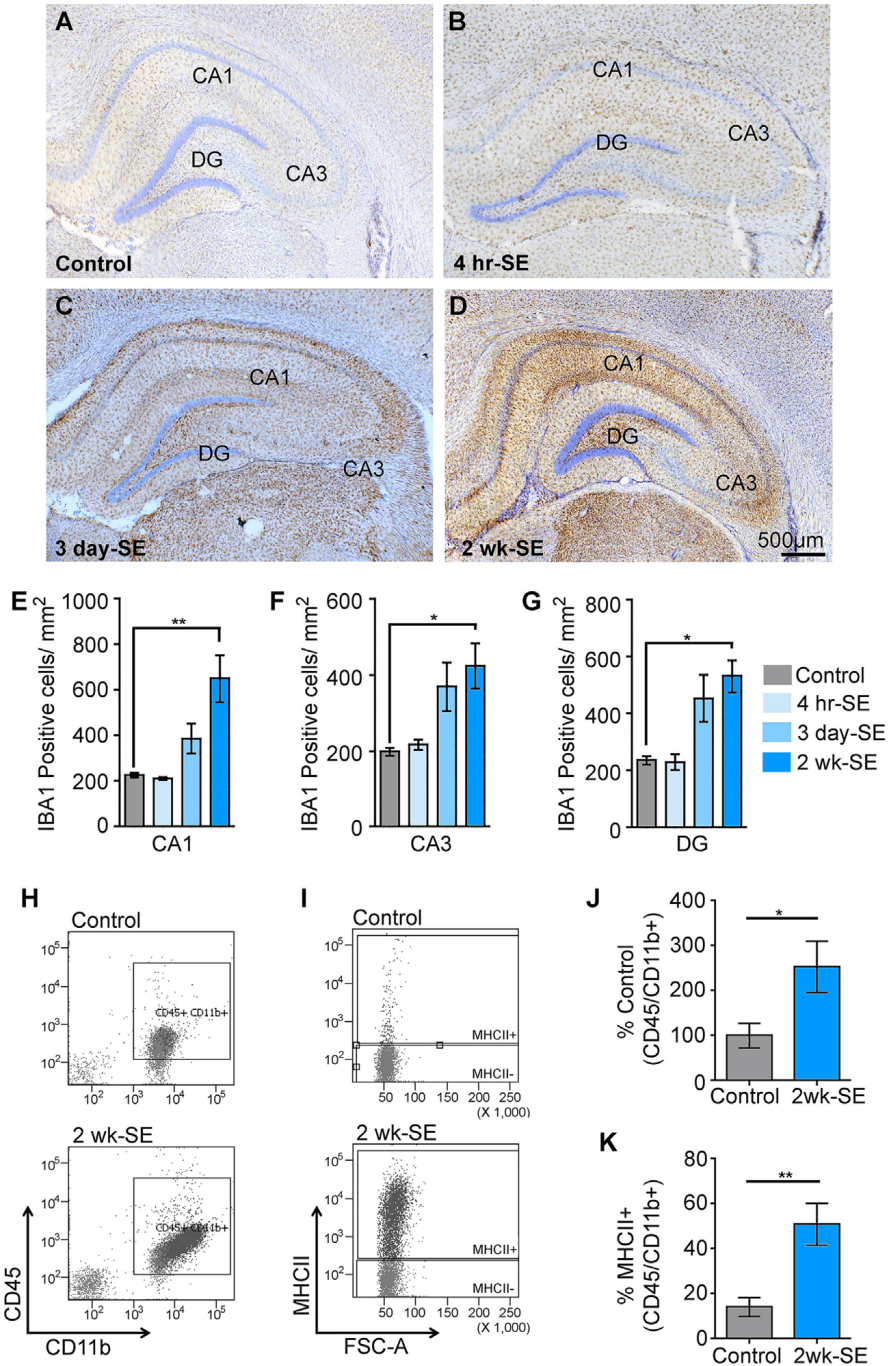
Status epilepticus (SE) provokes an increase in the number of microglia/macrophages in the hippocampus. **(A–D)** Representative images showing IBA1-postivibe immunoreactive cells in hippocampi from control **(A)** and SE groups at 4 h **(B)**, 3 days **(C)**, and 2 weeks **(D)** after the prolonged seizures. Nissl-stained nuclei are shown in blue. **(E–G)** Quantitative analysis of IBA1-positive cells in the hippocampal regions CA1 **(E)**, CA3 **(F)**, and dentate gyrus (DG) **(G)** from controls and SE groups. **(H–K)** Flow cytometry gating analysis of cluster of differentiation 45 (CD45) and cluster of differentiation molecule 11b (CD11b) **(H,J)**, and major histocompatibility complex class II (MHCII) **(I,K)** positive microglia/macrophages in hippocampal cell suspensions from control and 2-week-SE groups are shown **(J–K)**, show the analysis of the percentage of CD45/Cd11b+ positive cells **(J)** and from those the number of MHC11+ cell **(K)**. Data are shown as M ± SEM. *p < 0.05, **p < 0.002 by analysis of variance with Dunnett’s multiple comparison test.

We previously reported that a single episode of SE provoked an increase in the immunoreactivity for the microglial/macrophage marker IBA1 in the hippocampus that peaked at 2 weeks after the induction of SE (22). To further determine potential alterations in the population of IBA1-positive microglia/macrophages in the hippocampus, we counted the number of these cells in control samples as well as at 4 h, 3 days, and 2 weeks after SE (Figures 1A–G). Statistical analyses showed that the number of IBA1-positive cells in the hippocampal areas CA1, CA3, and DG was not different between the control group and the 4 h- or 3 day-SE groups (*p* > 0.05). Statistically significant differences were found between the control and 2-week-SE group. The highest SE-induced increases in the number of IBA1-positive cells at this time point occurred in the CA1 region (~1.9-fold, *p* = 0.0036) followed by DG (~1.3-fold, *p* = 0.0010) and CA3 (~1.2-fold, *p* = 0.0056) relative to control hippocampi. The increase in the abundance of microglia/macrophages at 2 weeks after SE was also evident through flow cytometry analyses (Figures 1H–K). Microglia/macrophages were identified with antibodies against CD45 and CD11b, and surface expression of the MHCII that was used to also label reactive/inflammatory cells (10). Figure 1H shows that whole hippocampal live-cell suspensions gated for CD45 and CD11b displayed significantly more labeled cells in the 2-week-SE samples than the controls (*p* = 0.0423) (Figures 1H,J). Similarly, a significant increase in the number of CD45/CD11b positive expressing MHCII was evident at the 2-week time point relative to controls (*p* = 0.0070) (Figures 1I,K). Together these data suggest that SE triggers an increase in the population of activated microglia/macrophages in the hippocampus.

**Figure 2:**
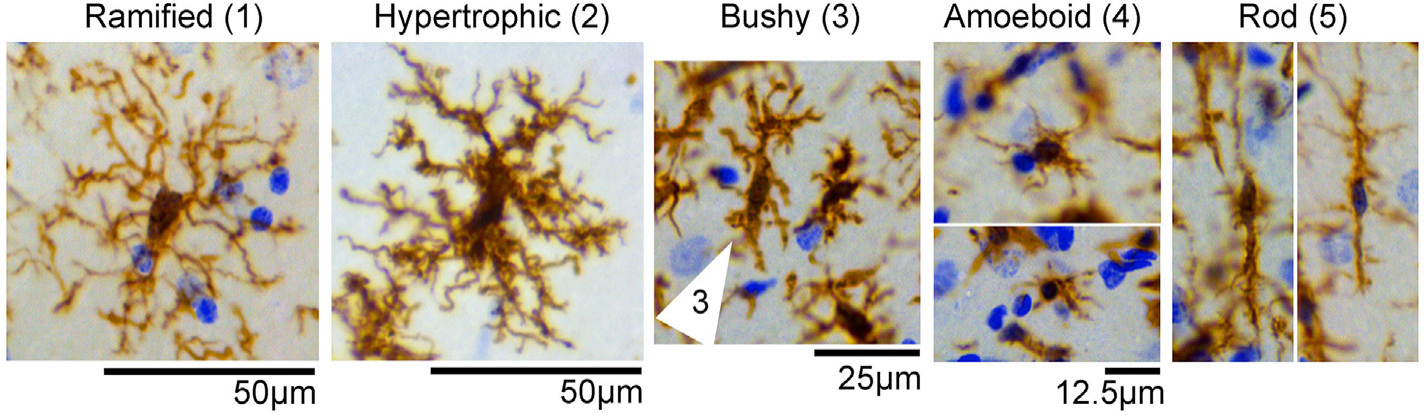
IBA1-positive cells display different morphologies in the hippocampus. Representative images of microglia/macrophage cells (brown) with different morphological phenotypes observed in control and status epilepticus (SE) groups included (1) ramified, (2) hypertrophic, (3) bushy (cell indicated by arrow), (4) amoeboid, and (5) rod. Images were taken from hippocampi of control or SE animals. Nissl-stained nuclei are shown in blue.

During the cell quantification analyses, we observed that IBA1-positive cells displayed a variety of heterogeneous morphologies, suggesting different activation and/or transitional states. We identified at least five different types of morphologies in the control and experimental SE tissues, and categorized them as follows: 1—ramified; 2—hypertrophic; 3—bushy; 4—amoeboid; and 5—rod (Figure 2). Some of these phenotypes such as 2- and 3- have been referred to as primed and reactive/activated, respectively (12). However, given that the function of these morphological phenotypes is not definitively known we based the nomenclature strictly on the physical appearance of the IBA1-positive cells.

**Figure 3:**
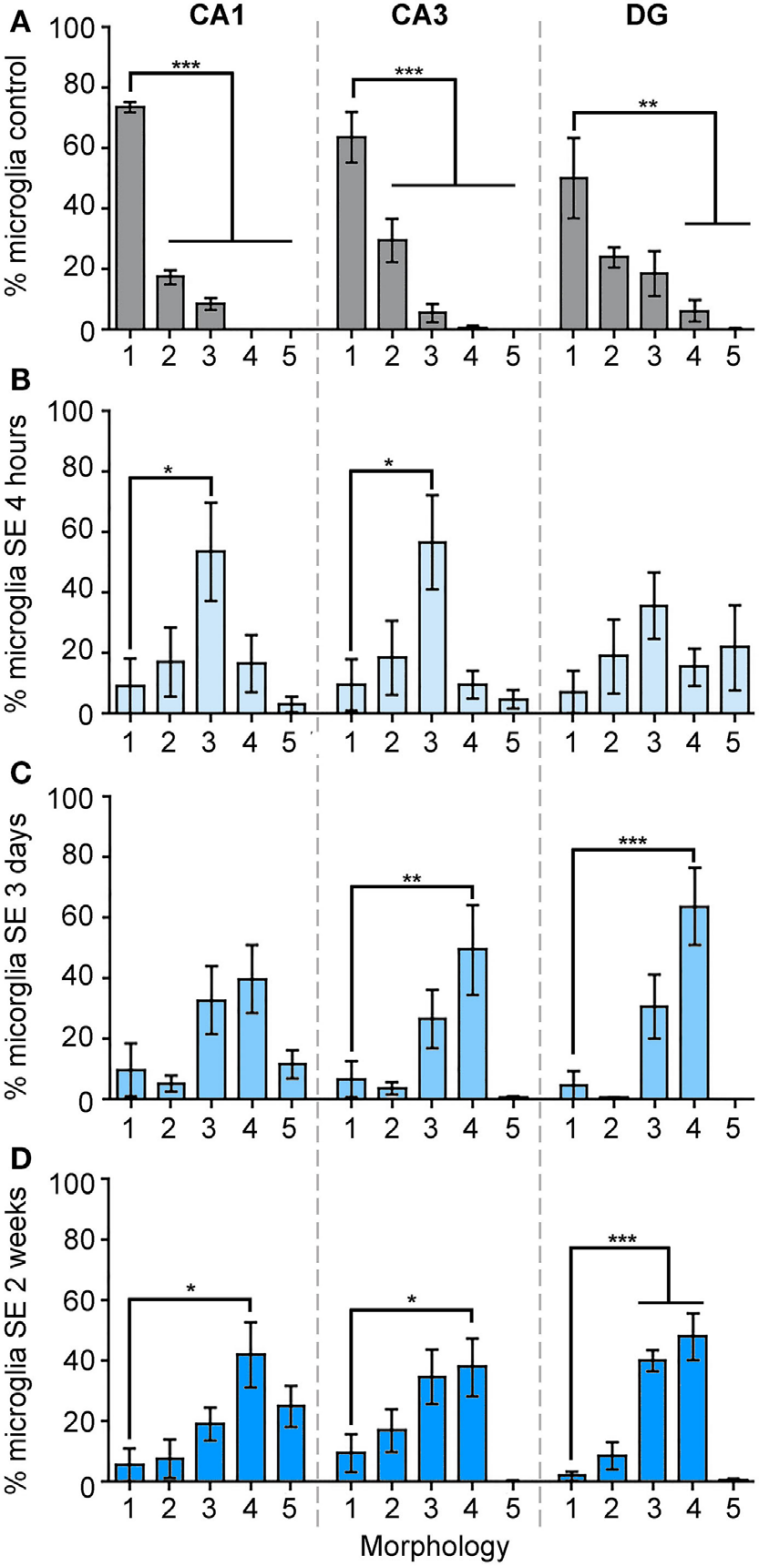
The morphology of IBA1-positive cells is mainly ramified in control hippocampi, and mainly bushy at 4 h, amoeboid at 3 days, and bushy/amoeboid at 2 weeks following status epilepticus (SE). Panels **(A–D)** show the analysis of the percentage of IBA1-positive cells of different morphologies: (1) ramified, (2) hypertrophic, (3) bushy, (4) amoeboid, and (5) rod, in the hippocampal CA1, CA3, and dentate gyrus areas in the control samples **(A)**, and in 4 h **(B)**, 3 days **(C)**, and 2 weeks **(D)** following SE samples. Data are shown as M ± SEM. *p < 0.05, **p < 0.001, ***p < 0.0001 by analysis of variance with Dunnett’s multiple comparison test.

We calculated the percentage of IBA1-positive cells that displayed each of these morphologies from the total cell numbers in the different hippocampal regions CA1, CA3, and DG in control brains, as well as at 4 h, 3 days, and 2 weeks (Figure 3; Figure S1 in Supplementary Material) after SE. Figure 3A shows that in the CA1 area of control hippocampi 74% of microglia were ramified with highly branched, thin, and elongated processes with an overall diameter of 40–50 µm, while 19% were hypertrophied and the remaining (~8%) were bushy. In CA3 area, 64% of the microglia displayed the ramified phenotype while 30% were hypertrophic, and in DG a mixed population of microglial/macrophage morphologies ranging from 1 to 4 was observed with 50% ramified, 24% hypertrophic, 19% bushy, and 6% amoeboid (Figure 3A).

Although the total microglia/macrophage numbers were similar in CA1 (*p* = 0.2777), CA3 (*p* = 0.2830), and DG (*p* = 0.8612) between the control and 4-h-SE groups (Figure 1), we found drastic changes in the microglia/macrophage morphology at this time point (Figure 3B). In the 4-h-SE group, the population of microglia/macrophage cells displayed mainly the bushy phenotype and represented 54% in CA1, 57% in CA3, and 36% in DG. Similarly, at 3 days after SE, the total number of IBA1-positive cells in the hippocampus was not significantly different to that of the control group (CA1, *p* = 0.0845; CA3, *p* = 0.0629; DG, *p* = 0.0654). However, the morphology of these cells was altered in all hippocampal regions of the 3-day-SE group (Figure 3C). In CA1, the bushy, amoeboid, and rod phenotypes contributed to 33, 40, and 12% of the population of IBA1-positive cells, respectively, in the 3-day-SE group. The bushy and amoeboid phenotypes also were the most abundant morphologies in the CA3 (27 and 50%, respectively) and DG areas (31 and 64%, respectively).

At 2 weeks after SE, the increase in the numbers of microglia in the hippocampus was significantly different when compared to control (Figure 1), and was coupled with a change in the morphology from 1—ramified to 2- through 5- phenotypes (Figure 3D). The distribution of the microglia/macrophage population in the 2-week-SE CA1 hippocampi was 6% ramified, 8% hypertrophic, 19% bushy, 42% amoeboid, and 25% rod. In the CA3 area of the 2-week-SE group, only 10% of the IBA1-positive cells were ramified while the larger population consisted of hypertrophic (17%), bushy (34%), and amoeboid (38%) with few rods (1%). In the DG, we observed a population that consisted of 2% ramified, 9% hypertrophic, 40% bushy, and 48% amoeboid cells.

**Figure 4:**
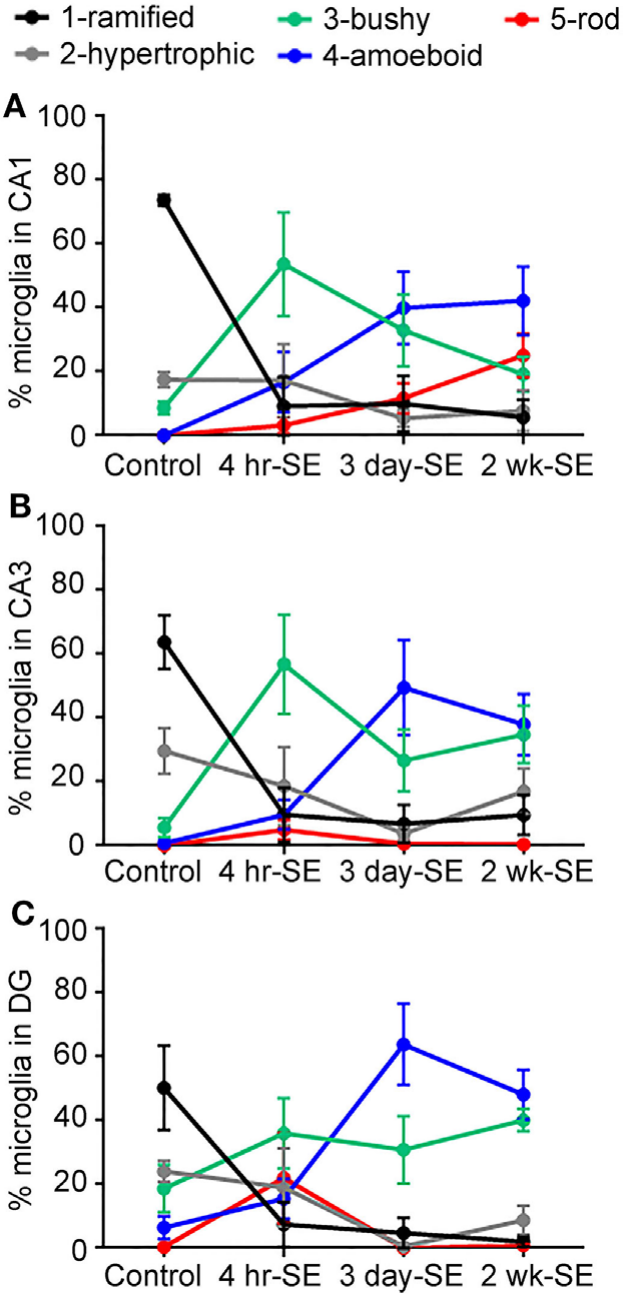
Status epilepticus (SE) triggers time-dependent changes in the morphology of IBA1-positive cells in the hippocampus. Panels **(A–C)** show the percentage of total hippocampal microglia/macrophage population of the different morphologies (1) ramified (black), (2) hypertrophic (gray), (3) bushy (green), (4) amoeboid (blue), and (5) rod (red) in the CA1 **(A)**, CA3 **(B)**, and dentate gyrus **(C)** hippocampal areas of control, 4-hr-SE, 3-day-SE, and 2-wk-SE groups. Note that the exact *p* values calculated from the statistical analyses of all data shown in this figure are described in the Section “Results.”

Overall, we found that the SE-induced changes in the microglia/macrophage morphological phenotype occurred as early as 4 hrs and persisted for at least 2 weeks after the prolonged seizures in all hippocampal areas (Figure 4). Statistical analyses between the control group and the 4-h-, 3-day-, and 2-week-SE groups showed a significant decrease in the population of ramified cells in CA1 (4 h/3 day: *p* = 0.0001; 2 week, *p* < 0.0001) (Figure 4A), CA3 (4 h, *p* = 0.0029; 3 day, *p* = 0.0001; 2 week: *p* = 0.0008) (Figure 4B), and DG (4 h, *p* = 0.0332; 3 day, *p* = 0.0028; 2 week: *p* = 0.0067) (Figure 4C). Across this time line, the bushy phenotype peaked at 4 h (Ctl vs. 4-h-SE, bushy: CA1, *p* = 0.0171; CA3, *p* = 0.0084; DG, *p* = 0.2202) and slightly decreased thereafter. The amoeboid phenotype was significantly more abundant in the 3day-SE (Ctl vs. 3-day-SE, amoeboid: CA1, *p* = 0.0185; CA3, *p* = 0.0268; DG, *p* = 0.0054) and 2-week-SE groups compared to controls (Ctl vs. 2-week-SE, amoeboid: CA1, *p* = 0.0043; CA3, *p* = 0.0047; DG, *p* = 0.0013), where few cells displayed that morphology. Interestingly, a significant increase in the percentage of rod microglia was only found in the CA1 area of the 2-week-SE group (Ctl vs. 2-week-SE, rod: CA1, *p* = 0.0063; CA3, *p* = 0.1436; DG, *p* = 0.3653). Taken together, these data support that SE induces time-dependent changes in microglia/macrophage morphology throughout the hippocampus.

## Discussion

The main findings of this study are that a single episode of SE triggers: (1) an increase in the number of microglia/macrophages in the hippocampus that is significantly different from controls at 2 weeks after SE (Figure 1); (2) time-dependent changes that are characterized by an increase in the population of microglia/macrophage with bushy shapes which peak at 4 h and are followed by an increase in the abundance of amoeboid cells at 3 days and 2 weeks after the prolonged seizures (Figures 3 and 4); and (3) a significant increase in the number of rod-shaped cells was only evident in the CA1 region at 2 weeks post-SE (Figures 3 and 4). Substantial evidence supports activation of microglial cells in humans and experimental epilepsy (4, 5). However, to our knowledge, this is the first study to perform a spatiotemporal analysis of the *population* of hippocampal IBA1-positive cells with different morphologies in the adult rat pilocarpine model of SE and acquired temporal lobe epilepsy.

Altered morphologies from the typical ramified phenotype, as well as expression and levels of cytokines and chemokines, have been widely used to identify microglial activation in human and experimental epilepsy, including in response to SE (17, 22, 24, 25, 27, 38, 39). For instance, using 3D reconstructions and morphometric analysis, Shapiro et al. (25) reported that following pilocarpine-induced SE, the IBA1-positive microglial cells in the adult hilus displayed different morphologies that included cells with larger and elongated cell bodies with thick and dense processes (hypertrophic), cells with thickened but complexed processes (bushy), and cells with rod cell body morphology at 1-, 3-, and 5-days, respectively, after the prolonged seizures (25). Consistent with their findings, we identified multiple microglia/macrophages with these morphologies in the DG area at all time points. We also found an increase in the amount of MHCII-positive cells at 2 weeks after SE suggesting a heterologous population of microglia with different M1/M2 polarization phenotypes at this time point (40). Furthermore, a comparable spatial and temporal profile of microglial morphological changes along with an increased population of microglia in the CA1 area were reported in the adult hippocampus following kainic acid and ischemic insults (41, 42) as well as microgliosis after corneal kindling and Theiler murine encephalomyelitis virus (26). Together, these studies, in different models that promote SE and epilepsy, suggest the possibility that neuronal hyperexcitability and injury may contribute to the microglial changes.

Similar to the adult brain, microglia/macrophages in the developing hippocampus also respond with evident morphological changes when exposed to SE (27, 43–45). For example, Patterson et al. (27) demonstrated that early-life SE induced by fever is associated with an acute increase in the abundance of amoeboid IBA1-positive cells, but not total microglia/macrophages, throughout the hippocampus. This was coupled to increased levels of TNFα (27). In the adult hippocampus, it is noticeable that the appearance of hypertrophic/bushy phenotypes parallels the time points when the cytokines TNFα and IL-1β were highest in the hippocampus after pilocarpine-induced SE (22, 24). Although the specific functional role of each of the morphologies is not definitively known, this observation suggests the possibility that hypertrophic/bushy microglia/macrophages may be associated with the production and release of inflammatory molecules.

One of the most interesting observations was the accumulation of rod-shaped microglial cells in the CA1 area of the 2-week-SE group (Figure 3). This type of microglia is also found in human brain samples associated with drug-resistant epilepsy (39). Interestingly, rod microglia have been found wrapped along apical dendrites of cortical neurons that were also surrounded by activated microglia in the human epileptic tissue samples (39). This type of interaction has also been reported in other human neurological and psychiatric disorders that include viral infections and dementia (11, 46). Evidence suggests that the interactions between rod microglia and dendrites also occur following brain injury (14, 47). While the physiological significance of rod-shaped microglia and their tight interactions with dendritic processes remains to be defined, it has been argued that rod microglia may be required to prevent further damage to injured areas (47). In addition, it is also possible that the microglia–dendritic contacts, independent of microglial morphology, may result in synaptic stripping (48–50). Recent studies support that microglia–dendritic interactions increase after SE (5–7, 23, 51). Interestingly, we reported a spatiotemporal correlation between increased IBA1 immunoreactivity, loss of Map2 immunostaining, and a reduction in dendritic spines in the CA1 hippocampus (22). These paralleled colocalization of multiple IBA1-labeled microglial processes with Map2-labeled CA1 apical dendrites 2 weeks following SE (23), when the population of microglia is mostly amoeboid and rod. Thus, we speculate that these microglia morphologies may contribute to the dendritic structural plasticity in CA1. However, whether these neuro–immune interactions are beneficial or detrimental for synaptodendritic stability after SE requires investigation.

Overall, the time course of the SE-induced microglia/macrophage morphological alterations and activation follows the classical descriptions of microglial plasticity from the ramified to the amoeboid morphology which may be associated with different functional roles (10, 15, 52). Ramified microglia are very dynamic and contribute to the physiology of developing and adult brains (8, 15, 52, 53). For instance, *in vivo* imaging studies demonstrated that ramified microglial cells are highly active, regularly survey their microenvironment, and make direct contacts with synaptodendritic structures in an activity-dependent manner (7, 8, 54, 55). Ramified microglia help maintain homeostasis in neuronal circuities by promoting, for example, dendritic spine growth (56), elimination of extranumerary synapses in developing networks (49, 50) as well as phagocytosis of the excessive apoptotic newborn cells in the hippocampal sub-ventricular zone (9, 28). Moreover, in response to immune disturbances, these cells alter their actin cytoskeleton to the different morphological phenotypes that include but are not limited to hypertrophic, bushy, and amoeboid. Based on our observations, the increased microgliosis seen at 3 days after SE (Figure 1) with bushy and amoeboid morphologies (Figure 3) paralleled an increase in the number of cleaved caspase 3-positive cells in CA1 hippocampus (22). This suggests the possibility that the microgliosis seen at 3 days after SE may be linked to the need for clearing the apoptotic cells. Although the amoeboid and bushy morphologies are often associated with inflammatory responses, as well as a high phagocytic capacity, the extent to which each of the morphologies relates to specific functions in the brain is still unclear. This is because most available evidence for morphology–function associations comes from *in vitro* cell culture studies (10, 15).

## Conclusion

This study shows that the abundance of microglia/macrophage of different morphologies (ramified, hypertrophic, bushy, amoeboid, and rod) in the hippocampus evolves between 4 h and 2 weeks following a single episode of SE. These data suggest that at different time points, distinct populations of microglia may serve specific functions, such as surveillance for ramified cells, perhaps inflammatory for hypertrophic and bushy at 4 h and 3 days after SE, and potentially highly phagocytic at the amoeboid stage (3 days and 2 weeks after SE). However, to determine these possibilities, comprehensive histological studies that directly identify their biochemical profile with specific inflammatory (M1/M2 polarization) (53) and phagocytic makers/indexes (57) are required. Substantial evidence supports that microglia activation plays a critical role in some of the neuropathology and pathophysiology associated with SE, and has been linked to the modulation of memory (58, 59), psychiatric disorders (60), and even suicide (61), all of which have been reported in epilepsy (62, 63). Studies using immunosuppressant drugs such as rapamycin, FK506, and minocycline, which modulate microglial activation (64), support that reactive microglia contribute to seizure-induced cell death and spine loss in the hippocampus as well as the development of spontaneous seizures and cognitive decline in some models of SE and epilepsy (23, 65–71). However, here, we show that there is a heterologous population of microglia of different morphologies throughout the hippocampus that can potentially promote neuroprotection and/or neurodegeneration to their surrounding neurons. Thus, understanding the morphologies of microglia, and their associated inflammatory/phagocytic phenotypes throughout the development of epilepsy after SE could allow for more targeted treatments focused on specifically altering the detrimental signals. This may provide different insights and potentially new directions in how we target and manage SE.

## Ethics Statement

This study was carried out in accordance with the recommendations of Institutional and NIH guidelines and Purdue Animal Care and Use Committee.

## Author Contributions

SW-J conducted induction of SE, perfusions, immunohistochemistry, imaging acquisition, analysis, performed cell counts along with densitometry analyses blinded to treatment groups, flow cytometry, and wrote the manuscript. SH conducted imaging acquisition, analysis, performed cell counts along with densitometry analyses blinded to treatment groups, flow cytometry, and wrote the manuscript. AB designed and initiated the project, and wrote the manuscript. SW-J, SH, and AB participated in the discussion of the experiments, data, and manuscript.

## Conflict of Interest Statement

The authors declare that the research was conducted in the absence of any commercial or financial relationship that could be construed as a potential conflict of interest.
