# Supplementary material for: Status Epilepticus Triggers Time-Dependent Alterations in Microglia Abundance and Morphological Phenotypes in the Hippocampus
Source: Front Neurol. 2017 Dec 18;8:700. doi: 10.3389/fneur.2017.00700 (PMC5741821; doi:10.3389/fneur.2017.00700)
Supplement: Supplementary file 1 [file Image_1.PDF]

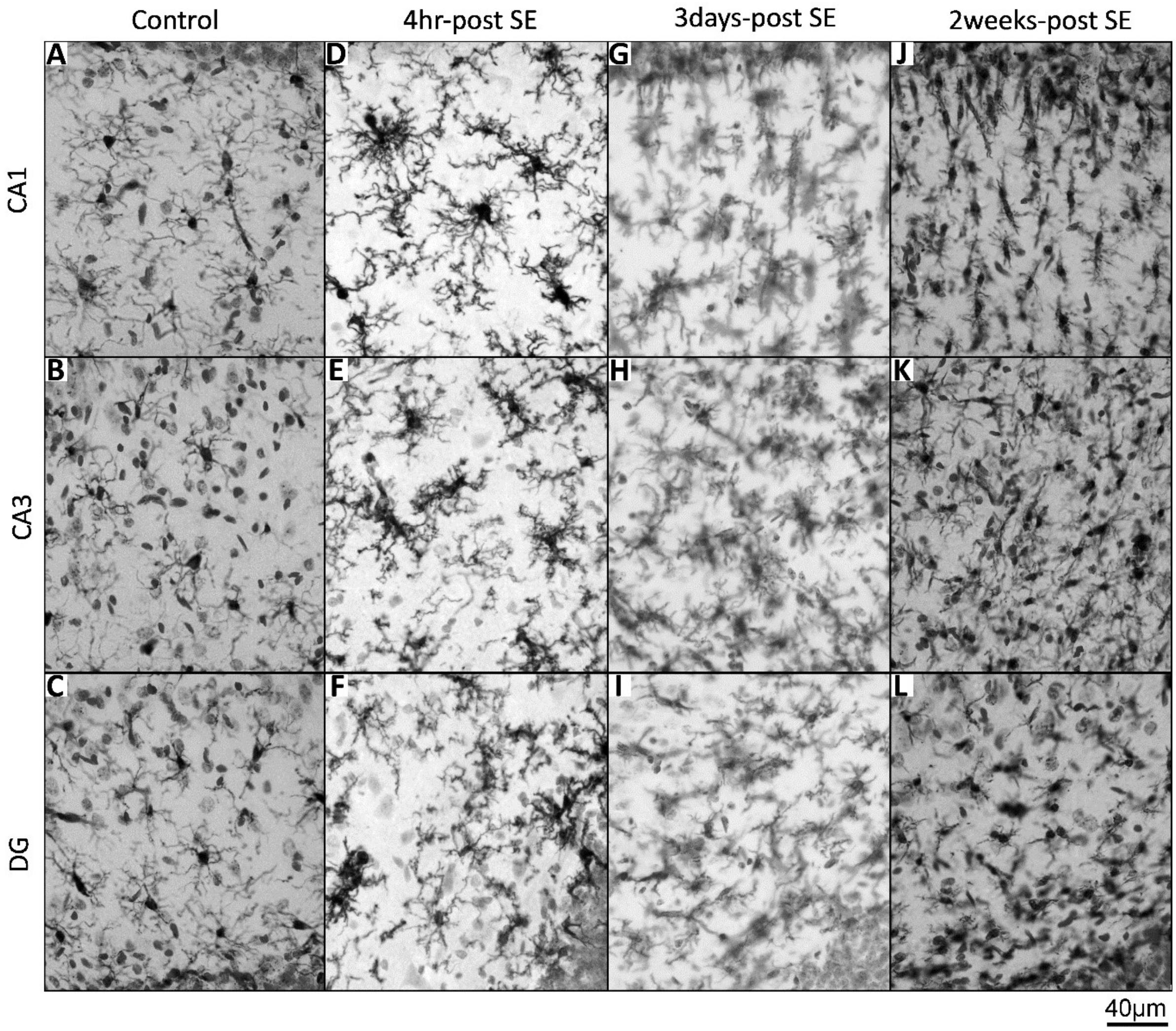

Supplemental Figure 1. The morphology of IBA1-positive cells is mainly ramified in control hippocampi. **A-F,** show the representative IBA1-positive cells (brown) images in the hippocampal CA1 (**A, D, G, J**), CA3 (**B, E, H, K**) and DG (**C, F, I, L**) areas in the control (**A-C**), 4hrs-post SE (**D-F**), 3days-post SE (**G-I**), and 2weeks-post SE (**J-L**) samples. Nissl stained nuclei are shown in blue.
